# Putative Adult Neurogenesis in Old World Parrots: The Congo African Grey Parrot (*Psittacus erithacus*) and Timneh Grey Parrot (*Psittacus timneh*)

**DOI:** 10.3389/fnana.2018.00007

**Published:** 2018-02-13

**Authors:** Pedzisai Mazengenya, Adhil Bhagwandin, Paul R. Manger, Amadi O. Ihunwo

**Affiliations:** School of Anatomical Sciences, Faculty of Health Sciences, University of the Witwatersrand, Johannesburg, South Africa

**Keywords:** doublecortin, proliferating cell nuclear antigen, old world parrots, Congo African grey parrot, Timneh grey parrot, adult neurogenesis, cell proliferation, cell migration

## Abstract

In the current study, we examined for the first time, the potential for adult neurogenesis throughout the brain of the Congo African grey parrot (*Psittacus erithacus*) and Timneh grey parrot (*Psittacus timneh*) using immunohistochemistry for the endogenous markers proliferating cell nuclear antigen (PCNA), which labels proliferating cells, and doublecortin (DCX), which stains immature and migrating neurons. A similar distribution of PCNA and DCX immunoreactivity was found throughout the brain of the Congo African grey and Timneh grey parrots, but minor differences were also observed. In both species of parrots, PCNA and DCX immunoreactivity was observed in the olfactory bulbs, subventricular zone of the lateral wall of the lateral ventricle, telencephalic subdivisions of the pallium and subpallium, diencephalon, mesencephalon and the rhombencephalon. The olfactory bulb and telencephalic subdivisions exhibited a higher density of both PCNA and DCX immunoreactive cells than any other brain region. DCX immunoreactive staining was stronger in the telencephalon than in the subtelencephalic structures. There was evidence of proliferative hot spots in the dorsal and ventral poles of the lateral ventricle in the Congo African grey parrots at rostral levels, whereas only the dorsal accumulation of proliferating cells was observed in the Timneh grey parrot. In most pallial regions the density of PCNA and DCX stained cells increased from rostral to caudal levels with the densest staining in the nidopallium caudolaterale (NCL). The widespread distribution of PCNA and DCX in the brains of both parrot species suggest the importance of adult neurogenesis and neuronal plasticity during learning and adaptation to external environmental variations.

## Introduction

Adult neurogenesis encompasses the birth and maturation of new neurons that become incorporated into existing circuitry or replace old and damaged neurons under normal physiological and or pathological conditions (Lindsey and Tropepe, 2006). The process of adult neurogenesis was confirmed in a variety of species from different taxa ranging from insects to humans (for reviews, see Cayre et al., 2002; García-Verdugo et al., 2002; Lindsey and Tropepe, 2006; Chapouton et al., 2007). Although this process has been confirmed in wide spectrum of species, the animals studied to date represent only a small fraction of the extant species, and studying further species will allow a broader understanding of the potential diversity of the process and function of adult neurogenesis (Barnea and Pravosudov, 2011). Across many species similarities in the process of adult neurogenesis have been observed, which include the origin of neurons, the phenotype of stem cells and their proliferative mechanisms, the migration and differentiation of neurons (for a review, see Doetsch and Scharff, 2001); however, they are also important differences among species in the spatial distribution of adult born neurons, their mode of migration and phenotypic diversity (Doetsch and Scharff, 2001; Ngwenya et al., 2017).

Numerous studies have revealed widespread adult neurogenesis in non-mammalian species such as fish, amphibians, reptiles, and birds when compared to mammalian species (García-Verdugo et al., 2002; Marchioro et al., 2005; Ghosh and Hui, 2016; Macedo-Lima et al., 2016; Ngwenya et al., 2017). Generally, the rate of adult neurogenesis decreases with age across vertebrate taxa (Kuhn et al., 1996; Knoth et al., 2010; Amrein et al., 2011; LaDage et al., 2011; Ngwenya et al., 2017), and the process is affected by factors such as genetics, endogenous and exogenous factors, and seasonal variation (for a review, see Barnea and Pravosudov, 2011).

African grey parrots are stocky, short tailed birds with an average body mass of 400 g. The Congo African grey parrot *(Psittacus erithacus)* is larger and lighter grey in color compared to the smaller and darker grey Timneh grey parrot *(Psittacus timneh)*. The African grey parrots live a long life span ranging between 20 and 50 years in the wild, but this can increase to nearly 100 years in captivity (Carey and Judge, 2001; Schmid et al., 2006). African grey parrots lead a complex social life, coupled with long term monogamous relationships (Seibert, 2006; Emery et al., 2007). In addition to displaying complex social interactions, African grey parrots are thought to exhibit potentially advanced cognitive capabilities comparable to Apes and young humans (Pepperberg et al., 1999; Emery and Clayton, 2004; Iwaniuk et al., 2004; Pepperberg, 2006). Their relative brain size is comparable to that of primates (Olkowicz et al., 2016). Parrots feature very high neuronal densities and high total neuronal numbers particularly in the forebrain when compared primates with larger brain sizes (Olkowicz et al., 2016). These parrots display vocal learning and mimicry abilities that rival those of young humans (Iwaniuk et al., 2004; Pepperberg, 2006) and these characteristics are valuable in studies examining and comparing vocal learning and cognitive abilities in avians and primates. Parrots show cooperative problem solving and the ability to discriminate discrete and continuous variables (Al Aïn et al., 2009; Péron et al., 2011). Here we investigated the generation and maturation of new neurons throughout the brains of the two subspecies of African grey parrots, the Congo African grey parrot (*Psittacus erithacus*), and the Timneh grey parrot (*Psittacus timneh*) using PCNA and DCX immunohistochemistry.

## Materials and Methods

### Animals and Tissue Processing

Two brains of adult male Congo African grey (*Psittacus erithacus*) and two brains of adult male Timneh grey parrots (*Psittacus timneh*) were used in the current study. The birds were purchased from a local breeder in South Africa, and were sacrificed and perfused in November 2013. The birds were treated and used according to the guidelines of the University of the Witwatersrand Animal Ethics Committee (clearance no: 2013/05/02B), which parallel those of the NIH for the care and use of animals in scientific experimentation. Five minutes prior to being euthanized, both birds were given an intramuscular dose of heparin, 2,500 units (0.5 ml) to prevent blood clotting. Animals were then injected with an intraperitoneal dose of Euthapent (1 ml/kg) and body mass recorded. The average body mass of the two Congo African grey parrots was 453.75 g, and the two Timneh grey parrots was 286.36 g. All animals were transcardially perfusion-fixed, initially with a rinse of 0.9% saline, followed by 4% paraformaldehyde in 0.1M phosphate buffer (PB, pH 7.4). The brains were carefully removed from the skull, and post-fixed overnight in 4% paraformaldehyde in 0.1M PB. The average brain mass of the Congo African grey parrots was 10.30 g and that of the Timneh grey parrot was 7.85 g. Before sectioning, the tissue was allowed to equilibrate in a 30% sucrose in 0.1M PB solution at 4°C for 4 days. The brains were then frozen in dry ice and sectioned in the coronal plane, 50 μm thick sections, on a sliding microtome. A one in ten series of sections were taken and three series stained for Nissl substance, PCNA and DCX. The remaining series of sections were placed in an antifreeze solution and are stored at -20°C for future use. The series of sections used for Nissl staining were mounted on 0.5% gelatine coated slides, dried overnight, cleared in a 1:1 mixture of 100% ethanol and 100% chloroform and stained with a 1% cresyl violet solution.

### Immunohistochemistry for PCNA and DCX

The series of sections used for free floating PCNA and DCX immunohistochemistry were initially treated for 30 min at room temperature under gentle shaking with an endogenous peroxidase inhibitor (49.2% methanol: 49.2% 0.1M PB: 1.6% of 30% H_2_O_2_ = 0.48% H_2_O_2_), followed by three 10 min rinses in 0.1M PB. To block non-specific binding sites the sections were then preincubated for 2 h, at room temperature under gentle shaking, in a blocking buffer solution consisting of 3% normal horse serum (NHS) for PCNA sections or 3% normal rabbit serum (NRS) for DCX sections, 2% bovine serum albumin, and 0.25% Triton X-100 in 0.1M PB. Following preincubation the primary antibodies were added to the blocking buffer solution and the sections were incubated for 48 h at 4°C under gentle shaking (PCNA – 1:500 dilution of mouse anti-PCNA, NCL-L-PCNA Leica Biosystems, Newcastle, United Kingdom; DCX – 1:300 dilution of goat anti-DCX antibody, C-18, Santa Cruz Biotechnology, Dallas, TX, United States) under gentle agitation. The primary antibody incubation was followed by three 10-min rinses in 0.1M PB and the sections were then incubated in a secondary antibody solution (PCNA sections – 1:1000 dilution of biotinylated anti-mouse IgG [BA-2001, Vector Labs] in 3% NHS and 2% bovine serum albumin in 0.1M PB; DCX sections – 1:1000 dilution of anti-goat IgG [BA-5000, Vector Labs] in 3% NRS and 2% bovine serum albumin in 0.1M PB) for 2 h at room temperature. This was followed by three 10-min rinses in 0.1 M PB, after which sections were incubated for 1 h in an avidin biotin solution (1:125 in 0.1M PB; Vector Labs, Burlingame, CA, United States), followed by three 10-min rinses in 0.1M PB. Sections were then transferred to a solution consisting of 0.05% diaminobenzidine tetrahydrochloride in 0.1M PB for 5 min at room temperature, after which 3.3 μl of 30% H_2_O_2_/ml of solution was added. With the aid of a low power stereomicroscope the progression of the staining was visually followed and allowed to continue until a level was reached where the background staining could assist in architectonic matching to the Nissl stained sections without obscuring the immunopositive structures. The tissue was then rinsed twice more in 0.1M PB before being mounted on glass slides coated with 0.5% gelatine and allowed to dry overnight. Once dry, the slides were placed in a solution of 70% ethanol for 2 h and then dehydrated, cleared in xylene and coverslipped with Depex. To test for non-specific staining of the immunohistochemical protocol, the primary and secondary antibodies were omitted from random sections and no staining was evident. The observed immunostaining patterns support the specificity of the antibodies and are compatible with observations made in pigeons, parakeets, and quails (Charvet and Striedter, 2008, 2009; Melleu et al., 2013, 2015).

### Analysis

The Nissl stained sections were examined with a low power stereomicroscope and the architectonic borders traced using a camera lucida. The PCNA and DCX immunostained sections were then matched to the drawings from the Nissl stained sections and the location of immunopositive soma marked on the drawings. Selected drawings were then scanned and redrawn using the Canvas 8 Software (Deneba Software, Miami, FL, United States). High power microscopic observation allowed for the determination of the relative densities of stained structures throughout the various regions of the brain. The relative densities of immunostained structures were visually compared and recorded on a scale ranging from low (+) to moderate (++) to high (+++). A second observer was used to eliminate observer bias.

The brain regions were identified and named in accordance with the stereotaxic atlas of the brain of the budgerigar (Brauth et al., 2011) using the nomenclature recommended by the Avian Brain Nomenclature Forum (Reiner et al., 2004). Digital photomicrographs were captured using a digital camera (Axio Cam HRc, Zeiss, South Africa) mounted on the light microscope (Axioskop 2 plus, Ziess, South Africa) and operating on the ZEN 2010 computer software (Zeiss, South Africa). No pixilation adjustments or manipulation of the captured images were undertaken, except for the adjustment of contrast, brightness, and levels using Adobe Photoshop 7.

## Results

### General Observations

**FIGURE 1:**
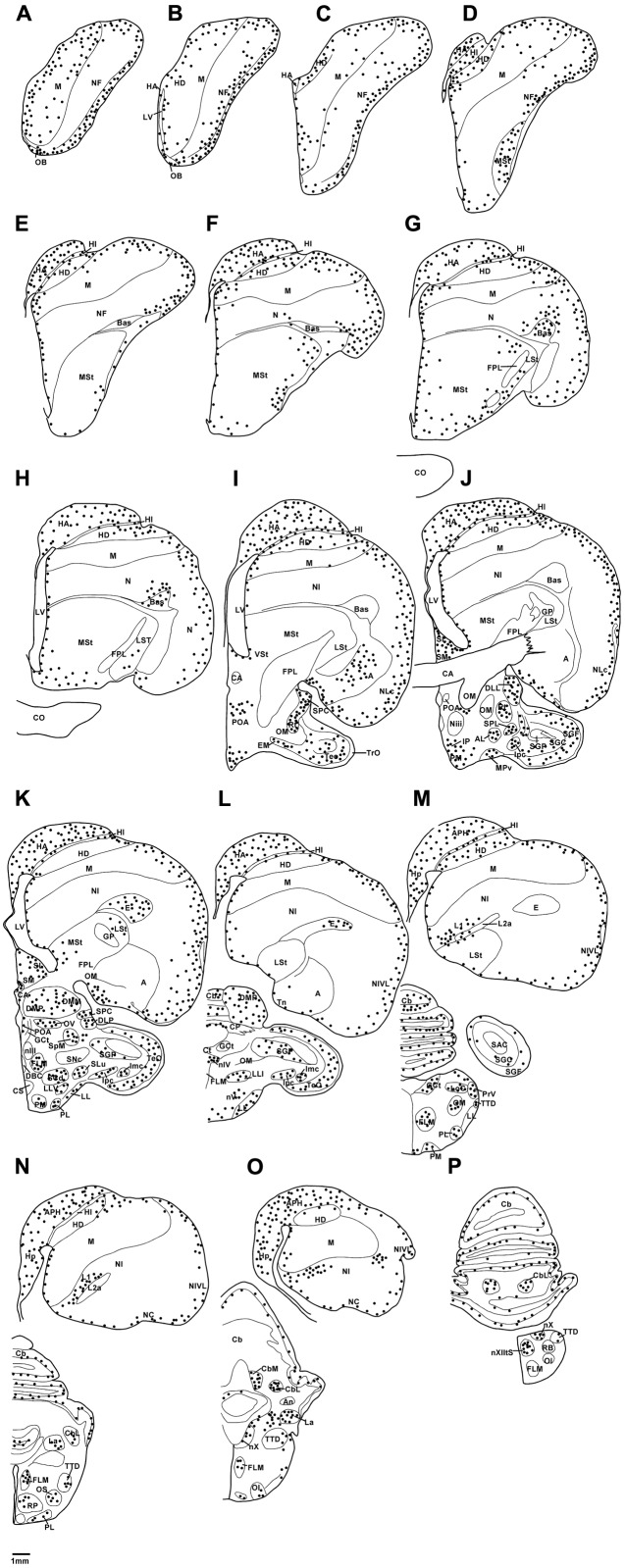
Diagrammatic reconstructions of a series of evenly spaced coronal sections through one half of the brain of the African grey Parrot illustrating the distribution of PCNA-immunoreactive cells. A single black dot indicates a single cell. Drawing **(A)** represent the most rostral section, **(P)** the most caudal, and each drawing is approximately 1,500 μm apart. For example distance between **A,B** is 1,500 μm. The same applies for **B–P**. See list for abbreviations.

**FIGURE 2:**
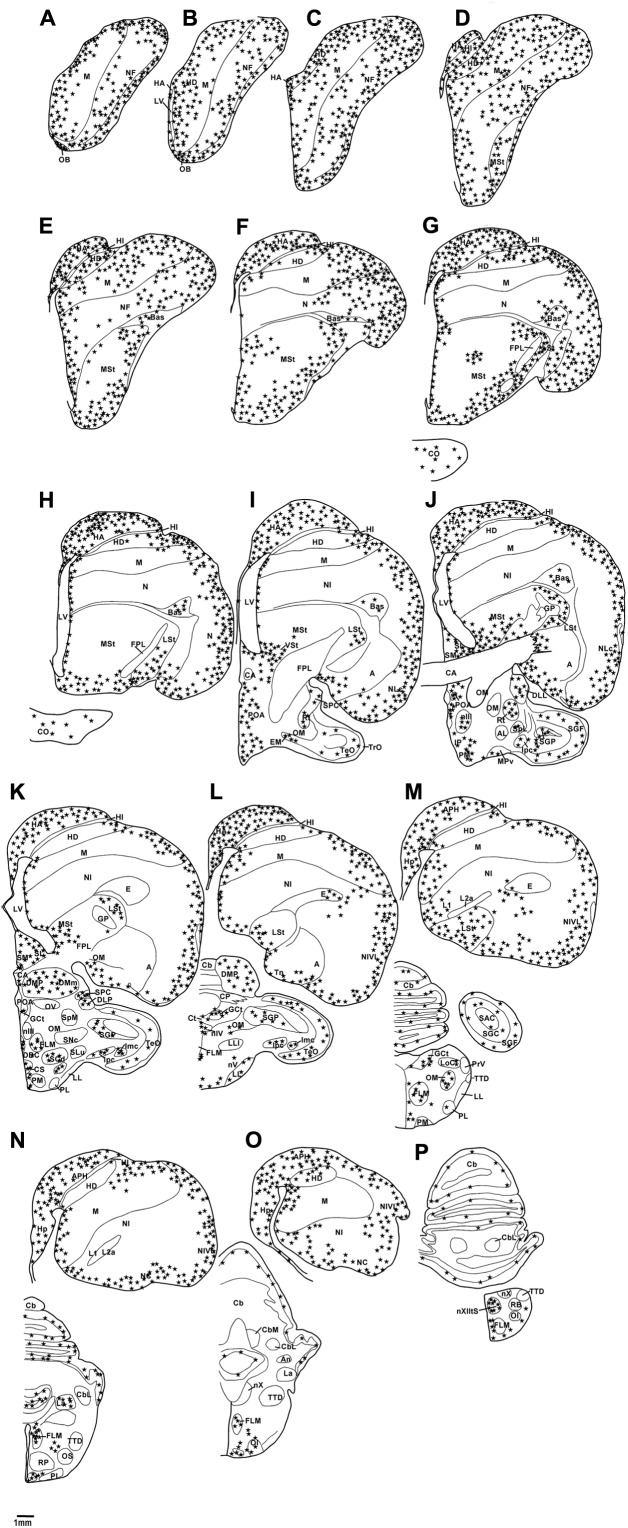
Diagrammatic reconstructions of a series of evenly spaced coronal sections through one half of the brain of the African grey Parrot, illustrating the distribution of DCX-immunoreactive cells. A single solid black star indicates a single cell body and a single open star represents a weakly stained cell. Drawing **(A)** represent the most rostral section, **(P)** the most caudal, and each drawing is approximately 1,500 μm apart. For example distance between **A,B** is 1,500 μm. The same applies for **B–P**. See list for abbreviations.

In the present study, we examined putative adult neurogenesis throughout the brains of the Congo African grey parrot *(Psittacus erithacus)* and the Timneh grey parrot *(Psittacus timneh)* using immunohistochemical techniques for the endogenous markers PCNA and DCX. The distribution of PCNA and DCX immunoreactivity was almost identical in both subspecies, but a few minor differences were observed (**Table 1**). Due to this extensive similarity we depict only the mapping of the distribution of the PCNA and DCX immunoreactive cells in the Timneh grey parrot (**Figures 1**, **2**). In both parrots, PCNA and DCX immunoreactivity was observed in the layers of the olfactory bulbs (OBs), the subventricular zone (SVZ) of the lateral, third and fourth ventricles and the cerebral aqueduct, subdivisions of the pallium (Hp, HA, HI, HD, M, N, E, and A), subpallium (MSt, LSt, SM, and SL), diencephalon, mesencephalon, and rhombencephalon. Generally, the telencephalic regions had a higher density of PCNA and DCX immunoreactive cells than other brain regions in both subspecies of parrots examined. In the majority of the telencephalic regions, the density of PCNA and DCX immunoreactive cells increased from rostral to caudal in both parrot subspecies. DCX immunoreactivity was more intense in fibers than in cell bodies and the majority of DCX immunopositive cells included small rounded cells, fusiform unipolar and bipolar cells.

**Table 1:** Summary of qualitative distribution and density of PCNA and DCX immunoreactive cells in the brain of the Congo African grey parrot and Timneh grey parrot.

Brain region		Congo African grey		Timneh grey	
			
		PCNA	DCX	PCNA	DCX
SVZ		**+++**	**+++**	**+++**	**+++**
OB		**+++**	**+++**	**+++**	**+++**
Telencephalon	HP	**++**	**++**	**++**	**++**
	HA	**++**	**++**	**++**	**++**
	HI	**++**	**++**	**++**	**++**
	HD	**++**	**++**	**++**	**++**
	M	**++**	**+**	**++**	**+**
	N	**+++**	**++**	**+++**	**++**
	NC	**++**	**+++**	**++**	**+++**
	E	**++**	**+**	**++**	**+**
	A	**++**	**+**	**++**	**+**
	MSt	**++**	**++**	**++**	**++**
	LSt	**++**	**++**	**++**	**++**
	GP	**-**	**-**	**-**	**-**
	SM	**+**	**+**	**+**	**+**
	SL	**++**	**+**	**++**	**+**
Diencephalon		**++**	**+**	**++**	**+**
Mesencephalon	Pretectum	**++**	**+**	**++**	**+**
	Optic tectum	**+++**	**+**	**+++**	**+**
Rhombencephalon	Cerebellum	**+**	**+**	**+**	**+**
	Pons	**++**	**+**	**++**	**+**
	Medulla oblongata	**++**	**+**	**++**	**+**


***-**, absent; +, low; ++, moderate; +++, high density of PCNA and DCX immunoreactive structures.*

### Distribution of PCNA Immunoreactivity

#### Olfactory Bulb

**FIGURE 3:**
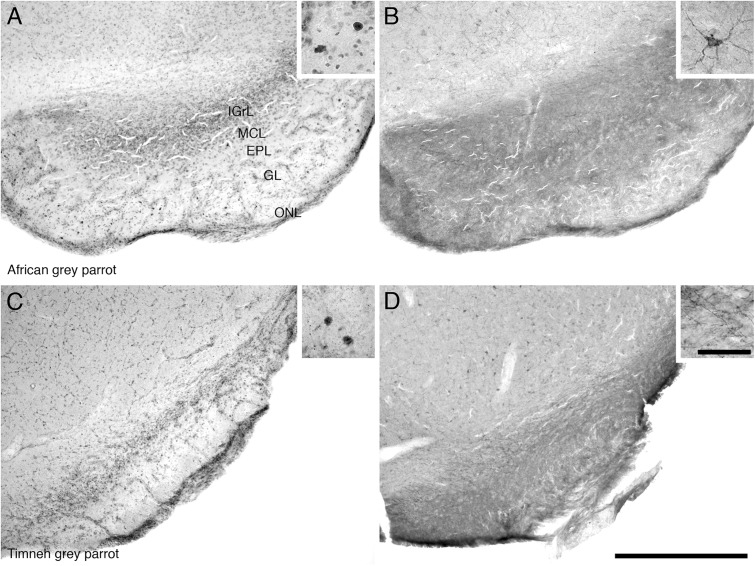
Photomicrographs of PCNA – **(A,C)** and DCX **(B,D)** – immunostained coronal sections through the olfactory bulb (OB) of the two parrot species – **(A,B)** – African grey parrot (*Psittacus erithacus*), **(C,D)** – Timneh grey parrot (*Psittacus timneh*). In all images dorsal is to the top and medial to the right. **(A,C)** PCNA immunoreactivity revealed a high number of PCNA-immunoreactive cells in the IGrL, MCL, and EPL layers of the olfactory bulb. Insets in **(A,C)** show a higher magnification images of PCNA immunoreactive cells. **(B,D)** DCX immunoreactivity revealed a high density of DCX-immunoreactive cells and fibers in all layers of the olfactory bulb (IGrL, MCL, EPL, and GL) except the olfactory nerve layer. Insets in **(B,D)** show a higher magnification of DCX immunoreactive cells and fibers. All high power images on inserts were taken from consecutive sections to low power images. Scale bar in **(D)** = 500 μm and applies to all. The scale bar in the inset in **(D)** = 50 μm and applies to all insets. See list for abbreviations.

Proliferating cell nuclear antigen immunoreactive cells were observed at high density in the IGrL, MCL, and EPL layer, while they were found in low density in the GL and ON layer (**Figures 3A,C**).

#### Subventricular Zone

**FIGURE 4:**
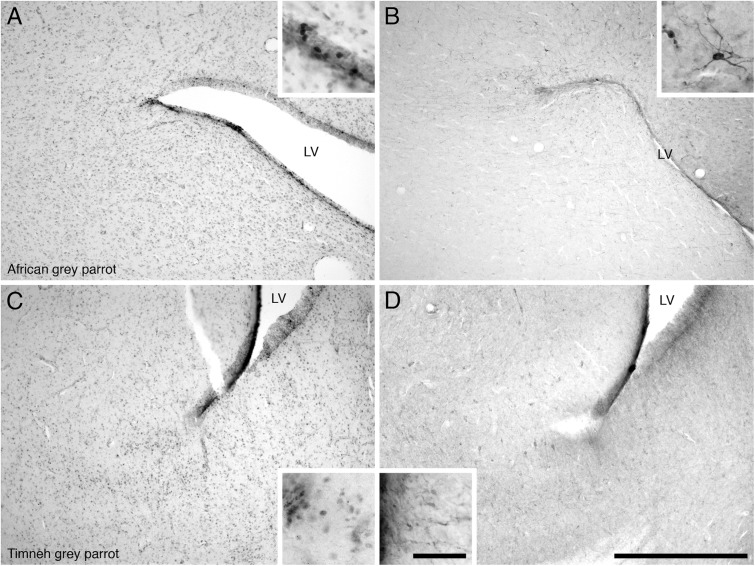
Photomicrographs of PCNA – **(A,C)** and DCX **(B,D)** – immunostained coronal sections through the subventricular zones (SVZ) of the lateral ventricle in the brains of the two parrot species – **(A,B)** – African grey parrot (*Psittacus erithacus*), **(C,D)** – Timneh grey parrot (*Psittacus timneh*). PCNA immunoreactivity revealed aggregates of proliferating cells (hot spots) in the SVZ of the dorsal pole of the lateral ventricle in the African grey parrot (*Psittacus erithacus*) **(A)**, and ventral pole of the lateral ventricle in Timneh grey parrot (*Psittacus timneh*) **(C)**. Insets in **(A,C)** show a higher magnification images of PCNA immunoreactive cells. DCX immunoreactivity revealed aggregates of DCX-immunoreactive cells and fibers in the SVZ of the dorsal pole of the lateral ventricle in the African grey parrot (*Psittacus erithacus*) **(B)**, and in the ventral pole of the lateral ventricle in the Timneh grey parrot (*Psittacus timneh*) **(D)**. Insets in **(B,D)** show a higher magnification of DCX immunoreactive cells and fibers. All high power images on inserts were taken from consecutive sections to low power images. In all images dorsal is to the top and medial to the right. Scale bar in **(D)** = 500 μm and applies to all. The scale bar in the inset in **(D)** = 50 μm and applies to all insets. LV, lateral ventricle.

In both parrot species the SVZ of the lateral ventricle exhibited a high density of PCNA immunoreactivity with occasional cells clustering. There was evidence of proliferative hot spots as described by Alvarez-Buylla et al. (1990) in the dorsal and ventral poles of the lateral ventricle in the Congo African grey parrots at rostral levels, but only a dorsal accumulation of PCNA immunoreactive cells was observed in the Timneh grey parrots (**Figures 4A,C**). The third and fourth ventricles and the cerebral aqueduct of both parrot species exhibited a medium density of PCNA immunoreactivity without cell clustering. There was a low density of PCNA immunoreactivity in the SVZ of the tectal portion of the cerebral aqueduct in both parrot species.

#### Pallial Regions

**FIGURE 5:**
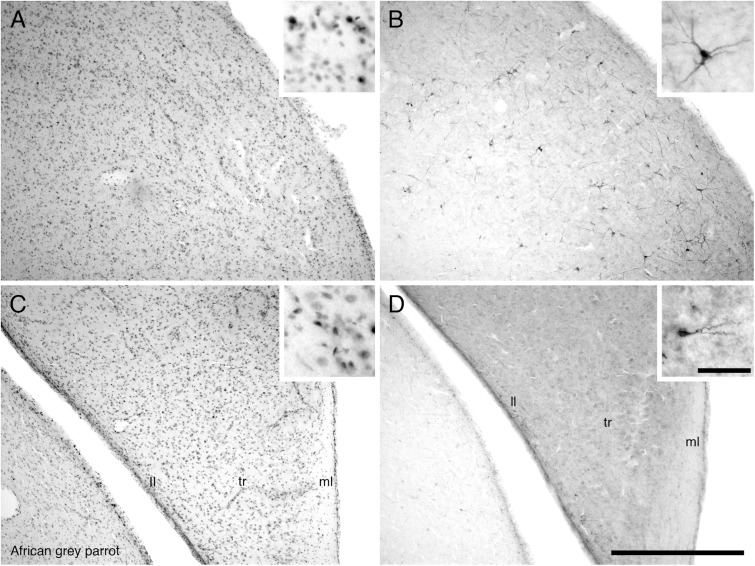
Photomicrographs of PCNA – **(A,C)** and DCX **(B,D)** – immunostained coronal sections through the hippocampus (HP) in the brain of the African grey parrot (*Psittacus erithacus*). Distribution of PCNA immunoreactive cells in the dorsal hippocampus **(A)**, and ventral layers of the hippocampus **(C)**. Insets in **(A,C)** show a higher magnification images of PCNA immunoreactive cells. Distribution of DCX immunoreactive cells in the dorsal hippocampus **(B)**, and ventral layers of the hippocampus **(D)**. Insets in **(B,D)** show a higher magnification of DCX immunoreactive cells. All high power images on inserts were taken from consecutive sections to low power images. Note multipolar DCX immunoreactive cells in **(B)** compared to unipolar DCX immunoreactive cells in **(D)**. In all images dorsal is to the top and medial to the right. Scale bar in **(D)** = 500 μm and applies to **(A–D)**. The scale bar in the inset in **(D)** = 50 μm and applies to all insets. See list for abbreviations.

In both parrot subspecies examined the HA, HD, HP, and APH exhibited a moderate density of PCNA immunoreactive cells (**Figure 5A**). In the ventral hippocampus the ml showed a low density of PCNA immunoreactive cells compared to the tr and ll which presented a moderate density (**Figure 5C**). The HI showed a low density of PCNA immunoreactive cells in the medial and core regions but an increase in the density of PCNA immunoreactive cells was observed in its lateral regions.

The mesopallium (M) exhibited a homogenous moderate density of PCNA immunoreactive cells at rostral levels, but at caudal levels the density of PCNA immunoreactive cells were reduced to low density in core regions, but moderate density in the medial and lateral regions. The entopallium exhibited a moderate density of PCNA immunoreactive cells and their distribution decreased markedly in caudal sections in both species of African grey parrots. There was a high density of PCNA immunoreactive cells in the nidopallium frontale (NF) of both parrot subspecies. The core region of NF of the Congo African grey parrot exhibited a large cluster of PCNA immunoreactive cells. At caudal levels, the NI and NC exhibited a moderate density of PCNA immunoreactive cells in the lateral regions, while the medial and core regions showed a low density of PCNA immunoreactive cells in both parrot species. The arcopallium exhibited a moderate density of PCNA immunoreactive cells which were distributed in higher density in the ventral regions encompassing the Tn than the dorsal regions, which showed a low density to almost no PCNA immunoreactive cells.

#### Subpallial Regions

In the striatum of both parrot species, a moderate density of PCNA immunoreactive cells was observed in MSt and LSt. PCNA immunoreactive cells were distributed in higher density in ventral regions of the MSt and also in regions adjacent to the ventrolateral wall of the lateral ventricle. No detectible PCNA immunoreactive cells were observed in the regions corresponding to the GP. Generally in the striatum, there was a rostro-caudal decline in the density of PCNA immunoreactive cells. In the septal complex of both parrots, the SM exhibited a low density of PCNA immunoreactive cells while the SL showed a moderate density of PCNA immunoreactive cells.

#### Diencephalon

**FIGURE 6:**
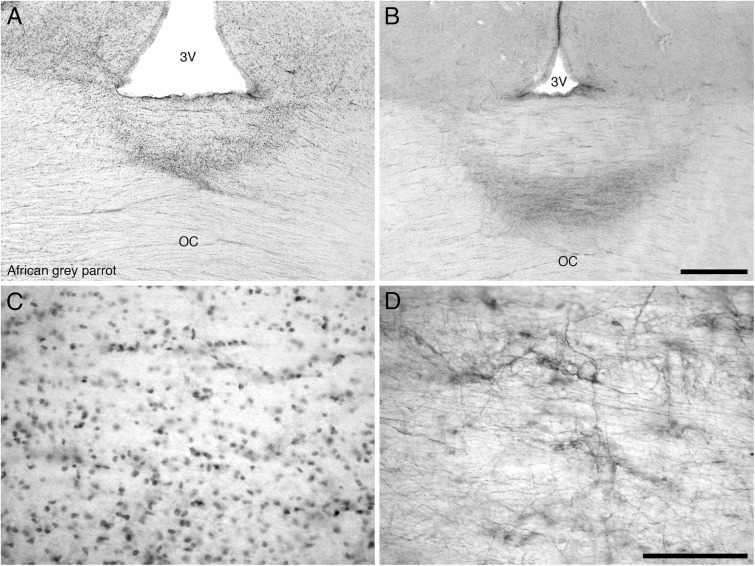
Photomicrographs of PCNA – **(A,C)** and DCX **(B,D)** – immunostained coronal sections through the optic chiasm (OC) at its border with the floor of the third ventricle (3V) in the brain of the African grey parrot (*Psittacus erithacus*). **(A)** Accumulation of PCNA immunoreactive cells in the optic chiasm (OC). **(C)** A high magnification image of the PCNA immunoreactive cells in the optic chiasm. **(B)** A concentration of DCX immunoreactive cells and fibers in the optic chiasm. **(D)** A higher magnification image of DCX immunoreactive cells and fibers showing distinct cells and thick fibers. These images depict a potential neurogenic zone at the dorsal midline of the optic chiasm. In all images dorsal is to the top. Scale bar in **(B)** = 500 μm and applies to **(A,B)**, scale bar in **(D)** = 100 μm and applies to **(C,D)**.

The diencephalon exhibited a high density of PCNA immunoreactive cells in the paraventricular nuclei, including POA and POM, the dorsal margin in the DMA, DLL, and DLP and the lateral margin in the nucleus rotundus (Rt), SPC, and SpL. In the core regions of the diencephalon, the African grey parrot exhibited a moderate density of PCNA immunoreactive cells in OV. The VMH in the ventral hypothalamus of both parrots exhibited the highest density of PCNA immunoreactive cells. The OC showed PCNA immunoreactive cells ventral to the inferior pole of the third ventricle in both species (**Figures 6A,C**).

#### Mesencephalon, Rhombencephalon, and Cerebellum

Most of the regions of the midbrain exhibited a moderate density of PCNA immunoreactive cells, including the nIII, GCt, IP and the isthmic region in the SLu, Ipc, and Imc. In the optic tectum in both parrots a higher density of PCNA immunoreactive cells was observed in the SGF and SGC.

**FIGURE 7:**
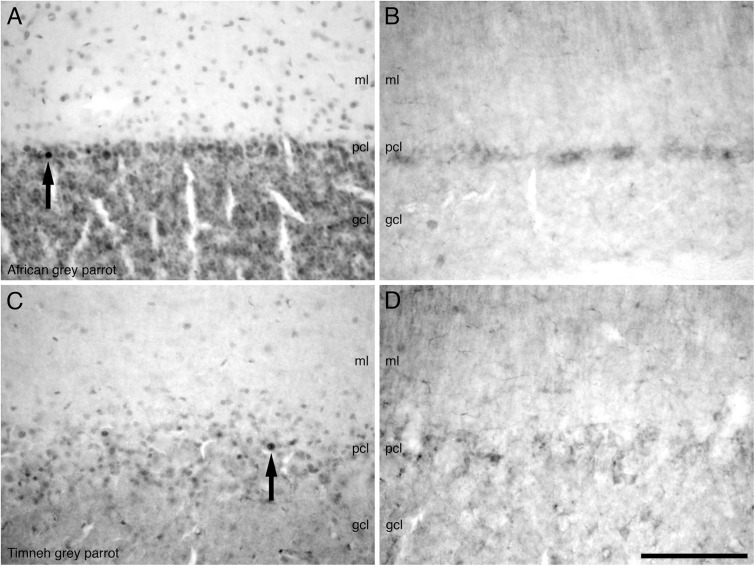
Photomicrographs of PCNA – **(A,C)** and DCX **(B,D)** – immunostained coronal sections through the cerebellar cortex in the brains of the two parrot species – **(A,B)** – African grey parrot (*Psittacus erithacus*), **(C,D)** – Timneh grey parrot (*Psittacus timneh*). A clear similarity in the distribution of PCNA immunoreactive cells in the layers of the cerebellar cortex in the African grey parrot **(A)** and Timneh grey parrot **(C)** is observed. A similar distribution and staining intensity of DCX immunoreactive cells and fibers in the layers of the cerebellar cortex in the African grey parrot (*Psittacus erithacus*) **(B)** and Timneh grey parrot (*Psittacus timneh*) **(D)** is also observed. Note large and deeply stained PCNA immunoreactive cells (*arrows*) in the Purkinje cell layer (pcl) in both parrots. In all images dorsal is to the top and medial to the right. Scale bar in **(D)** = 100 μm and applies to all. See list for abbreviations.

In the cerebellum, a moderate density of PCNA immunoreactive cells was observed in the Purkinje cell layer and the CbM and CbL (**Figures 7A,C**). Occasional PCNA immunoreactive cells were observed in the molecular and granule cell layers. In the pons and the medulla oblongata, a moderate density of PCNA immunoreactive cells was observed in some regions not limited to the LoC, medial and lateral pontine nuclei (PM and PL), PrV, TTd, and nucleus raphe (R).

### Distribution of DCX Immunoreactivity

#### Olfactory Bulb

The OB was the region that contained the highest density of DCX immunoreactive structures in both parrots. DCX immunoreactive cells and fibers were seen in high density in the inner layers of the olfactory bulb, but in lower density in the outer layers (**Figures 3B,D**).

#### Subventricular Zone

In both parrot subspecies, the SVZ of the lateral ventricle exhibited a high density of DCX immunoreactive cells and fibers. The majority of the DCX immunoreactive cells were orientated parallel to the walls of the lateral ventricles, depicting tangential migration, while a few DCX immunoreactive cells were oriented perpendicular to the walls of the lateral ventricle, suggesting radial migration. At some rostral levels in the Congo African grey parrot there were occasional accumulations of DCX immunoreactive cell clusters in the dorsal and ventral poles of the lateral ventricle, similar to the dorsal and ventral ‘hot spots’ suggested by Alvarez-Buylla et al. (1990) (**Figures 4B,D**). Such accumulations of DCX immunoreactive cells were only observed in the dorsal pole of the lateral ventricle of the Timneh grey parrots. The SVZ of the third and fourth ventricles and that of the cerebral aqueduct showed a low density of DCX immunoreactive fibers with no cells clearly discernable.

#### Pallial Regions

The pallial portion of the telencephalon exhibited the highest density of DCX immunoreactive cells and fibers, although the density of DCX immunopositive structures varied across different pallial regions in both parrots. Moderate densities of DCX immunoreactive cells and fibers were present in the HA, HD, medial and lateral NF, nidopallium (N), and NI. The medial and lateral regions of the HI and the M showed a low density of DCX immunoreactive cells and fibers. There was almost no DCX immunoreactivity detected in the core of HI and M. In the entopallium a low density DCX immunoreactive cells and fibers was observed. At rostral levels of these telencephalic pallial regions, DCX immunoreactive cells and fibers were present in a slightly higher density laterally than medially. Generally there was a rostro-caudal decrease in the amount of DCX immunoreactive material in these pallial regions. The NC and the nidopallium caudolaterale (NCL) exhibited a much higher density of DCX immunoreactive cells and fibers than any other pallial region, particularly in the ventral and lateral aspects. The arcopallium exhibited a very low density of DCX immunoreactive cells and fibers in both parrot species. The HP and APH contained a moderate density of DCX immunoreactive cells and fibers (**Figure 5B**). In the ventral hippocampus, a moderate density DCX immunoreactive material was observed in the tr and ll while the ml showed a low density (**Figure 5D**).

#### Subpallial Regions

The medial and lateral striatum exhibited a moderate density of DCX immunoreactive cells and fibers at rostral levels, but the density of these cells and fibers decreased noticeably at levels caudal to the anterior commissure (CA). In the MSt, the DCX immunoreactive cells and fibers were observed in higher density in the medial and ventral aspect than in the dorsal and lateral aspects. A low density of DCX immunoreactive cells and fibers was observed in the SM and SL; however, at caudal levels in both parrots the SL showed a moderate density of DCX immunoreactive cells and fibers. No detectible DCX immunoreactive structures were observed in the regions corresponding to the GP.

#### Diencephalon

The diencephalon exhibited a very low density of DCX immunoreactive cells and fibers. A weak DCX immunoreactivity was observed in the paraventricular nuclei, including POA and POM of the medial hypothalamus and in the VMH of the ventral hypothalamus and in the dorsolateral group of nuclei of the diencephalon, which include the DMA, DLL, DLP, nucleus rotundus (Rt), SPC, and SpL. The OC showed DCX immunoreactive fibers (**Figures 6B,D**), while no staining was detected in CA.

#### Mesencephalon, Rhombencephalon, and Cerebellum

A low density of DCX immunoreactive fibers was observed in the layers of the optic tectum (SGF and SGC). Some mesencephalic nuclei and tracts also exhibited weak DCX immunoreactive fibers and vesicles including the GCt, nIV, ICo, and the fasciculus longitudinalis medialis (FLM). In the cerebellum, the Purkinje cell layer was weakly stained, with only fibers being observed (**Figures 7B,D**). In the pons and medulla oblongata weakly stained fibers and vesicles in very low density were observed in some nuclei including, but not limited to, the LLV, PM, PL, La, raphe nucleus (R), nXIIts, and nX.

## Discussion

### General Considerations

In the current study we examined putative adult neurogenesis in the brains of the two species of African grey parrots the Congo African grey parrot (*Psittacus erithacus*) and Timneh grey parrot (*Psittacus timneh*) using PCNA and DCX immunohistochemistry. The PCNA immunoreactive cells were distributed heterogeneously in all brain regions including the olfactory bulb, telencephalon, diencephalon, mesencephalon and rhombencephalon, while DCX immunoreactive structures were dense in the olfactory bulb, telencephalon and some regions of the diencephalon, but far less dense in some parts of the diencephalon, the mesencephalon and rhombencephalon in both African grey parrot species. To a large extent our results conform and are comparable to the wide spread distribution of adult neurogenesis reported in other birds (Kim et al., 2006; Boseret et al., 2007; Balthazart et al., 2008; Vellema et al., 2014; Mazengenya et al., 2017). Despite the overall similarity in the distribution of PCNA and DCX in both African grey parrot species, region specific differences, particularly associated with the density of PCNA and DCX immunoreactive structures, were observed in the telencephalon. Such region specific differences might highlight the relationship between adult neurogenesis and brain function, particularly in regions responsible for certain behavioral repertoires in different species.

### Validity of PCNA and DCX Antibodies in Determining Adult Neurogenesis in Birds

The PCNA antibody used in the current study recognizes the 36 kDa nuclear protein subunit of the DNA polymerase that is essential for DNA replication and is expressed selectively in proliferating cells (Hall et al., 1990; Balthazart and Ball, 2014). The PCNA antibody is present during the various stages of the cell cycle except the G_0_ stage (Kurki et al., 1988; Hall et al., 1990; Balthazart and Ball, 2014). In birds, the PCNA antibody has been characterized in developing quail, parakeets, zebra finches (Charvet and Striedter, 2008, 2009) and chickens (Hannan et al., 1999; Capes-Davis et al., 2005). The regions where PCNA immunoreactivity was observed in the African grey parrots correspond with those reported in quails after BrdU injection (Charvet and Striedter, 2008). Apart from labeling proliferating cells, the PCNA antibody also labels cells repairing their DNA (Hall et al., 1990; Shivji et al., 1992; Essers et al., 2005) and remains detectable several days after cells exit the cell cycle (Balthazart and Ball, 2014). Such characteristics can affect the reliability of the PCNA antibody as a marker of adult neurogenesis in birds since they lead to an overestimation of proliferating cells.

Doublecortin is a microtubule associated protein that plays a key role in the migration of neurons during development and in post mitotic neurons undergoing migration, remodeling of their dendritic processes, and synaptogenesis in adulthood (Francis et al., 1999; Gleeson et al., 1999; Nacher et al., 2001; Brown et al., 2003; Yang et al., 2004; Capes-Davis et al., 2005; Couillard-Despres et al., 2005). DCX expression levels have been found to decline with age in mammals (Brown et al., 2003; Couillard-Despres et al., 2005) and in birds (Ling et al., 1997; Hannan et al., 1999; Capes-Davis et al., 2005; Kim et al., 2006). In rats DCX expression is first observed 1 day after cell division and last for about 2–3 weeks until the neurons begin to express markers of mature neurons such as NeuN (Brown et al., 2003). In birds, DCX expression has been found to last for up to 30 days post mitosis (Balthazart et al., 2008). The DCX antibody was recommended for adult neurogenesis (Rao and Shetty, 2004; Couillard-Despres et al., 2005) and its expression has been consistent in areas known to recruit new neurons in mammals (Brown et al., 2003). In contrast, DCX expression in birds has been found in locations not associated with adult neurogenesis. In adult canaries BrDU immunoreactive cells were found to co-express the DCX antibody in the subtelencephalic regions (Vellema et al., 2014). The DCX antibody used in the current study has been characterized in the following birds: canaries (Boseret et al., 2007; Balthazart et al., 2008; Yamamura et al., 2011; Vellema et al., 2014), chickadees (Fox et al., 2010), zebra finches (Kim et al., 2006), sparrows (LaDage et al., 2010), starlings (Hall and MacDougall-Shackleton, 2012), pigeons (Melleu et al., 2013, 2015; Mazengenya et al., 2017) Japanese quail (Balthazart et al., 2010) and chickens (Hannan et al., 1999; Capes-Davis et al., 2005; Mezey et al., 2012).

### Adult Neurogenesis in African Grey Parrots Compared to Other Birds

Adult neurogenesis has been observed in budgerigars (*Melopsittacus undulatus*), which are small parrots (Nottebohm, 2011), but the current study is the first to report the presence of adult neurogenesis in the brains of the larger African grey parrots. There are remarkable similarities and as well as some variations in the pattern of adult neurogenesis across the avian species studied to date. In the avian brain, new neurons are generated in the SVZ of the lateral ventricle, with the largest density of proliferating cells reported in its dorsal and ventral poles. These cells migrate to different target regions of the brain (Alvarez-Buylla et al., 1994). In the present study proliferating cells were observed in high density in the SVZ of the lateral ventricle and throughout its rostrocaudal extent. The Congo African grey parrot exhibited an accumulation of proliferating cells in the dorsal and ventral poles of the lateral ventricle, at the level of CA, similar to the proliferative “hot spots” described by Alvarez-Buylla (1990). These aggregates of proliferating cells were only observed in the dorsal pole of the lateral ventricle in the Timneh grey parrot. The proliferative hot spots phenomenon was also reported in other adult birds, including pigeons (Melleu et al., 2013), canaries (Alvarez-Buylla, 1990; Alvarez-Buylla et al., 1998; Mazengenya et al., 2017), chickens (Mezey et al., 2012), and marsh tits (*Poecile palustris*) (Patel et al., 1997); however, they were absent in the adult ring dove (*Streptopelia risoria*) (Ling et al., 1997). The identity of the proliferating cells in these germinal zones remains contentious in both mammals and non-mammals. In the song bird, *Serinus canaria* the proliferative hot spots were associated with large accumulations of radial glial cells (Alvarez-Buylla and Nottebohm, 1988; Alvarez-Buylla et al., 1988), suggesting that radial glial cells were the primary cells undergoing mitosis in the germinal zones (Alvarez-Buylla et al., 1990; Goldman, 1990; Vellema et al., 2010). In contrast, cell proliferation, including in the present study, was observed in the brain parenchyma (Alvarez-Buylla et al., 1988; Vellema et al., 2010; Mazengenya et al., 2017), the SVZ of the cerebral aqueduct, third, fourth, and tectal ventricles, albeit in low density, and in the midbrain, hindbrain and cerebellum (Vellema et al., 2010). This localized proliferative activity suggests that certain neurons may be produced and integrate locally without the need for cell migration, and or thus proliferating cells can be glia or endothelial cells (Vellema et al., 2010).

The recruitment of new neurons is widespread in the telencephalon of adult birds and affected by various factors including age, environmental complexity, seasonal variation, hormones, stress and social complexity (for review, see Barnea and Pravosudov, 2011). In the African grey parrots, the distribution of DCX immunoreactive cells and fibers exhibited a similar pattern to that observed in the adult canaries (Boseret et al., 2007; Balthazart et al., 2008; Vellema et al., 2014), starlings (Absil et al., 2003) and zebra finches (Kim et al., 2006); however, DCX immunoreactivity was reported to be restricted to the telencephalon in other studies on adult canaries (Alvarez-Buylla and Nottebohm, 1988; Kirn et al., 1994; Vellema et al., 2010), pigeons (Melleu et al., 2013), ring doves (Ling et al., 1997), and chickens (Mezey et al., 2012).

Neuronal recruitment exhibited regional differences in the telencephalic subdivisions of adult canaries, zebra finches, domestic pigeons, and rock pigeons (Kim et al., 2006; Boseret et al., 2007; Balthazart et al., 2008; Melleu et al., 2013; Mazengenya et al., 2017). In the adult canaries and zebra finches, the highest densities of DCX immunoreactive cells were observed in the M, NC, and striatum. Intermediate DCX expression was observed in the hyperpallium and rostral nidopallium, while a low density of DCX immunopositive cells was noted in the hippocampus, arcopallium and subtelencephalic regions (Kim et al., 2006; Boseret et al., 2007; Vellema et al., 2014). We observed similar trends in the adult African grey parrots, but the hippocampus and parahippocampal regions exhibited moderate to high density of DCX immunoreactive cells and fibers. In addition, DCX immunoreactive neurons and fibers showed some area specific distribution in certain regions of the telencephalon and the brain stem nuclei in the two species of African grey parrots. For example, rostral nidopallium exhibited a moderate density DCX immunoreactive cells and fibers, whereas the caudal nidopallium exhibited the densest DCX immunoreactive cells and fibers. In addition, the distribution of DCX immunoreactive cells was not uniform in most telencephalic regions, where DCX immunoreactive cells and fibers were found in higher density in the medial and lateral margins compared to the core regions. The area of specific distribution of DCX immunoreactive cells and fibers observed in these parrot species was also reported in adult canaries (Vellema et al., 2010, 2014) and might highlight brain areas where plastic changes are necessary for the maintenance of behavioral repertoires in different species. In both song and non-song birds, the arcopallium and the entopallium exhibited absent to low densities of DCX immunoreactive cells (Goldman and Nottebohm, 1983; Vellema et al., 2010; Melleu et al., 2013; Mazengenya et al., 2017). Similarly, the two species of African grey parrots showed a low density of DCX immunoreactive cells in the entopallium and the arcopallium. In the arcopallium, the DCX immunoreactive cells and fibers were distributed only in the ventrocaudal margins encompassing the nucleus taenia (Tn).

The morphology of DCX immunoreactive cells varied in the different regions of the brain in the two species of African grey parrots. In regions closer to the lateral ventricle particularly the ventral striatum the DCX immunoreactive cells exhibited elongated somata with unipolar or bipolar morphology. In other regions of the telencephalon elongated DCX immunoreactive cells were mixed with cells with round and multipolar morphology, although the former were more abundant. Elongated unipolar or bipolar cells represent migrating young neurons, usually emanating from the SVZ, whereas the round and multipolar neurons characterize mature neurons (Alvarez-Buylla, 1990). Migration of neurons in the telencephalon of birds follows two defined modes including tangential and radial migration (Alvarez-Buylla et al., 1988; Alvarez-Buylla, 1990). Vellema et al. (2010) further described the random migration of neuroblasts, whereby migrating immature neurons follow undefined routes within the telencephalon. In birds, the most pronounced mode of migration is radial migration, whereby migrating neuroblasts are guided on cellular processes of radial glial cells (Alvarez-Buylla and Nottebohm, 1988; Alvarez-Buylla, 1990; Vellema et al., 2010). Under this mode of migration, young neurons migrate from the SVZ of the lateral ventricle preferentially toward the lateral and ventrocaudal aspects of the telencephalon (Alvarez-Buylla, 1990; Vellema et al., 2010). In birds including in the current study, proliferative hot spots, both dorsal and ventral (PCNA immunoreactive as in the present study) coincide with accumulations of immature and migrating neurons (DCX immunoreactive cells and fibers) particularly in the hyperpallium and the striatum, respectively (Alvarez-Buylla and Nottebohm, 1988; Alvarez-Buylla, 1990). According to Alvarez-Buylla (1990), radial glia processes extend laterally for about 2 mm into the parenchyma of the adult avian telencephalon and these radial cells are concentrated in proliferative hot spots suggesting that migration through scaffolds of the radial glia processes is only possible for very short distances. In the current study, the Congo African grey parrot showed accumulation of a higher density of DCX immunoreactive cells and fibers in regions of the telencephalon corresponding to the dorsal and ventral hot spots whereas in the Timneh grey parrot a higher density of DCX immunoreactive structures was observed in the dorsal regions of the telencephalon.

This preferred direction of migration may explain the presence of the high densities of DCX immunoreactive cells and fibers in the lateral and caudal regions of the telencephalon when compared to the medial and rostral regions. Migration of immature neurons furthest from the extends of the radial glia processes becomes random and neuroblasts migrate and differentiate in regions that are topographically disjointed from the proliferative hot spot indicating that there is no SVZ specification for the final destination and position where new neurons differentiate and integrate (Alvarez-Buylla and Nottebohm, 1988; Alvarez-Buylla, 1990). This may also help to explain the variations in the topography of the proliferative hot spots observed in the current study and in other birds examined to date. According to Melleu et al. (2013), the majority of round and multipolar cells expressing the microtubule marker DCX do not co-express with the adult neuronal marker NeuN, suggesting that they are immature neurons even though they show the morphology of mature neurons.

Recruitment of new neurons in the diencephalon, mesencephalon and rhombencephalon of adult birds has been reported in experimental animals (Cao et al., 2002; Chen et al., 2006), but no studies support adult neuronal recruitment under normal physiological conditions. Despite this, reports in adult canaries (Boseret et al., 2007; Balthazart et al., 2008; Vellema et al., 2014) and zebra finches (Kim et al., 2006) indicate the presence of DCX immunoreactive cells in these regions. In the species of the African grey parrots studied herein, similar, but low density, distributions of DCX immunoreactive cells and fibers were observed. In agreement with Kim et al. (2006) and Boseret et al. (2007) the low density expression of DCX immunoreactivity in the brain stem may not signify adult recruitment of new neurons, but represent cellular plasticity of adult neurons (Nacher et al., 2001; Brown et al., 2003).

### Functional Implications of Adult Neurogenesis Related to Certain Behaviors of the African Grey Parrots

The role of adult neurogenesis in brain function and behavior in various species is still contentious. In mammals adult hippocampal neurogenesis is generally associated with acquisition of new and clearance of old memories (Kempermann et al., 2004; Zhao et al., 2008); however in birds, neuronal recruitment is wide spread in the telencephalon, making it difficult to understand the precise functional implications of this widespread characteristic. In addition, birds live comparably longer than mammals of similar body mass, and adult neurogenesis in avian species may be adaptive, promoting continuous learning by updating and renewing memories (Nottebohm, 1991; Gahr et al., 2002). The effect of learning on adult neurogenesis is bidirectional. Learning has been found to increase the survival, maturation and the response to stimuli of adult born neurons (Kee et al., 2007; Tronel et al., 2010; Lemaire et al., 2012). In contrast, adult neurogenesis facilitates learning in new conditions, which may include new habitats, new members of social groups, or new sources of food. In avian species adult neurogenesis is coupled with cell death (Kirn and Nottebohm, 1993; Rasika et al., 1994; Yamamura et al., 2011) and has been found to be task-dependant in various species (Barker et al., 2011). According to Nottebohm et al. (1986) increased singing in canaries leads to increased neuronal recruitment in the high vocal centre (HVC). In food storing species increased hippocampal neurogenesis was observed during the peak periods of food caching in autumn, whereas in non-storing species no addition of hippocampal neurons was recorded during the same period (Barnea and Nottebohm, 1996; Sherry and Hoshooley, 2009). Moreover in migratory species, which rely on spatial memory, species with larger home ranges exhibited increased hippocampal neurogenesis compared to related species that inhabit smaller territories (Cristol et al., 2003; Hoshooley and Sherry, 2007; LaDage et al., 2009). The olfactory bulbs recruit new neurons in adults of almost all studied species, except for a few studies of canaries and zebra finches (Barker et al., 2011; Melleu et al., 2013). In the current study we observed a high density of PCNA and DCX immunoreactive structures in the inner layers of the olfactory bulbs in both African grey parrots. The functional implications of olfactory neurogenesis remain elusive, but the olfactory system is involved in reproduction, the monitoring of environmental changes, the maintenance of survival by detecting presence of predators, the identification of clan members and the location of food (Jones and Roper, 1997; Patzke et al., 2010; Barker et al., 2011).

Apart from adult hippocampal and olfactory neurogenesis, neuronal recruitment in the NC has been shown to increase under various states of environmental enrichment. In a study conducted on zebra finches, adult neurogenesis in the NC was found to increase in adults introduced to communal living compared to individuals living in pairs (Lipkind et al., 2002). The reason associated with this increase is the possibility that individuals introduced to communal living need to identify new members and establish new social relationships (Lipkind et al., 2002; Adar et al., 2008). Similar changes in neuronal recruitment in the NC were identified during the reproductive cycle, when adult individuals of zebra finches were feeding their young (Barkan et al., 2007). In the present study, the NC and the NCL exhibited the densest DCX immunoreactivity in both species of African grey parrots. The NC is associated with reproductive behavior (Melleu et al., 2013), whereas the NCL, a proposed analog of the mammalian prefrontal cortex, participates in higher order cognitive functions, such as decision making, speech and planning of behavior (Güntürkün, 2005).

African grey parrots live in large colonies of approximately 10,000 individuals (Parr and Juniper, 2010). Although few studies have examined the behavior of African grey parrots in the wild, results show that their social ecology is comparable to that of primates (Emery et al., 2007). The African grey parrots lead a complex social life coupled with long term monogamous relationships (Seibert, 2006; Emery et al., 2007). Shultz and Dunbar (2010) postulated that species in monogamous relationships develop larger brain to body mass ratios to preserve the stable pair bonded relationships. The African grey parrots may be useful models to assess the impact of changing social environments on adult neurogenesis.

## Author Contributions

AI and PRM designed the study, analyzed the data, and reviewed the final manuscript for submission. PM and AB collected and processed tissue and carried out the initial analysis of data. PM prepared the initial manuscript draft.

## Conflict of Interest Statement

The authors declare that the research was conducted in the absence of any commercial or financial relationships that could be construed as a potential conflict of interest.

## Abbreviations

A: arcopallium
AL: ansa lenticularis
An: nucleus angularis
APH: area parahippocampalis
Bas: nucleus basorostralis
CA: commissura anterioris
Cb: cerebellum
CbL: nucleus cerebellaris lateralis
CbM: nucleus cerebellaris medialis
OC: optic chiasm
CP: commissura posterior
CS: nucleus centralis superior (Bechterew)
DBC: brachium conjunctivum decendens
DCX: doublecortin
DLL: nucleus dorsolateralis anterior thalami pars lateralis
DLP: nucleus dorsolateralis posterior thalami
DMA: nucleus dorsomedialis anterior thalami
DMM: magnicellular nucleus of the dorsomedial thalamus
DMP: nucleus dorsomedialis posterior thalami
E: entopallium
EM: nucleus ectomammillaris
EPL: external plexiform layer of the olfactory bulb
FPL: fasciculus prosencephali lateralis
GCt: substantia grisea centralis
GL: glomerular layer of olfactory bulb
GP: Globus pallidus
HA: hyperpallium apicale
HD: hyperpallium densocellulare
HI: hyperpallium intercalatus
HP: hippocampus
IGrL: internal granular layer of the olfactory bulb
Imc: nucleus isthmi, pars magnocellularis
IP: nucleus interpeduncularis
Ipc: nucleus isthmi pars parvocellularis
L1: lamina 1 of Field L
L2a: lamina 2a of Field L
La: nucleus laminaris
ll: lateral layer of the ventral hippocampus
LLV: ventral nucleus of the lateral lemniscus
LoC: locus coeruleus
LSt: lateral striatum
LV: lateral ventricle
M: mesopallium
MCL: mitral cell layer of the olfactory bulb
ml: medial layer of ventral hippocampus
MPV: nucleus mesencephalicus profundus
MSt: medial striatum
N: nidopallium
NC: nidopallium caudale
NI: nidopallium intermedium
nIII: nucleus oculomotorius
NIVL: nidopallium intermedium pars ventrolateralis
NLc: central nucleus of the lateral nidopallium
nV: nucleus motorius nervi trigemini
nX: vagus nucleus
nXIIts: nucleus nervi hypoglossis pars tracheosyringealis
OI: nucleus olivaris inferioris
OM: tractus occipitomesencephalicus
ON: olfactory nerve
OV: nucleus ovoidalis
PCNA: proliferating cell nuclear antigen
PL: nucleus pontis lateralis
PM: nucleus pontis medialis
POA: nucleus preopticus anterior
POM: nucleus preopticus medialis
PrV: nucleus sensorium principalis nervi trigemini
RP: nucleus reticularis pontis
Rt: nucleus rotundus
SCv: nucleus subcoeruleus ventralis
SGC: stratum griseum centrale
SGF: stratum griseum et fibrosum
SL: lateral septum
SLu: nucleus semilunaris
SM: medial septum
SNc: substantia nigra pars compacta
SPC: nucleus superficialis parvocellularis
SpM: nucleus spiriformis medialis
SpL: nucleus spirformis lateralis
TeO: Optic tectum
tn: *nucleus taenia* of the amygdala tr, triangular area of ventral hippocampus
TrO: Optic tract
TTd: nucleus et tractus descendens nervi trigemini
Tu: nucleus tuberis
VMH: ventromedial hypothalamus nucleus
VSt: ventral striatum
